# Horses’ attentional characteristics differ according to the type of work

**DOI:** 10.1371/journal.pone.0269974

**Published:** 2022-07-25

**Authors:** Céline Rochais, Mathilde Stomp, Mélissa Sébilleau, Mathilde Houdebine, Séverine Henry, Martine Hausberger

**Affiliations:** CNRS, UMR 6552 −Laboratoire Ethologie Animale et Humaine -EthoS-, Station Biologique, Université de Rennes, Université de Caen-Normandie, Paimpont, France; Massey University, NEW ZEALAND

## Abstract

Attention is a central process of cognition and influences the execution of daily tasks. In humans, different types of work require different attentional skills and sport performance is associated with the ability to attention shift. Attention towards humans varies in dogs used for different types of work. Whether this variation is due to the recruitment of individuals suitable for specific types of work, or to the characteristics of the work, remains unclear. In the present study, we hypothesized that domestic horses (*Equus caballus*) trained for different types of work would also demonstrate different attentional characteristics but we also explored other possible factors of influence such as age, sex and breed. We exposed more than sixty horses, working in 4 different disciplines, and living in two types of housing conditions, to a visual attention test (VAT) performed in the home environment. Individual attentional characteristics in the test were not significantly influenced by age, sex, breed or conditions of life but were strongly related to the type of work. Riding school horses showed longer sequences and less fragmented attention than all other horses, including sport horses living in the same conditions. Interestingly, sport performance was correlated with attention fragmentation during the test in eventing horses, which may need more attention shifting during the competitions. Working conditions may influence attention characteristics indirectly through welfare, or directly through selection and training. Our study opens new lines of thought on the determinants of animal cognition and its plasticity and constitutes a further step towards understanding the interrelationship between working conditions and cognition.

## Introduction

Both perceptual and cognitive processes are limited in capacity [1]. As a result, the cognitive function of attention is the ability to selectively process one relevant item of the environment over others [1]. Attention is considered as a central process in stimuli perception and filtering before undergoing extensive processing in the brain [2]. Attention is at the core of cognition [3] and influences the execution of daily tasks such as reading the newspaper, driving or working. As a consequence, jobs recruiters may favour persons with particular attentional abilities in attention. For example, piloting of aircraft is multifaceted: it demands high vigilance, energetic arousal and concentration [4]. Thus, pilot’s attentional skills are commonly evaluated with attentional battery of tests [5]. Alternatively, the demands of working activities impact attention performances. Some studies have combined behavioural observation and brain activity (measured with electroencephalography–EEG, or functional near-infrared spectroscopy—fNIRS), and have shown reduced activation in prefrontal and motor brain regions after one hour of work on repetitious assembly tasks (such as in ‘assembly line’ work), suggesting attentional decrease [6–8]. Other studies have revealed the difficulty of switching attention between tasks or showing fragmentation of attention (*i*.*e*., fragmentation of attention: pattern of attention related to the ability to shift attention from one stimulus to another and to the frequency with which a subject pay attention to a particular stimulus [9]) between multiple tasks (*e*.*g*., talking to a client while writing an email to another person, managing transition among different projects, [10]). But, long-term training in managing multiple tasks has been shown to improve cognitive processing. For example, increased training in basket-ball athletes is associated with improved performances in a multiple attention task suggesting an increase of attentional load [11] *i*.*e*., amount of attentional resources toward the stimulus [12]. Attentional shifting and updating were also positively correlated with sports performance in elite soccer players [13].

Some of domestic animals are also involved in working activities. As in humans, animals could be selected for a particular type of work based on their attentional abilities and/or the type of work they are trained for, can have over the time an impact on their attention performances [14]. While some scientific studies have attempted to identify the individual attentional characteristics of working dogs that make them suitable for specific work tasks (*e*.*g*., agility, rescue, assistance) [15], studies testing the possibility that work *per se* influence attentional characteristics are scarce. Some of them explored pseudo-social attentional characteristics: for example, dogs trained in tasks requiring cooperation with a handler (*e*.*g*., agility, rescue) gazed more at humans than untrained dogs [16, 17], whereas dogs trained for more independent roles (*e*.*g*., guide dogs) gazed less than untrained dogs [18, 19]; agility dogs shifted gaze to the owner more frequently than assistance and untrained dogs [14].

In domestic horses, individual differences in attentional characteristics have been described and it appeared that visual attention in an experimental task (visual attention task, “VAT”), was predictive of attention during a working task (lunge task) [20]. Other studies suggest that work conditions may influence attentional performance: training with positive reinforcement enhances attention towards the trainer and the task [21, 22], trainers’ own attention influences horses’ attention [23, 24]. Furthermore, the type of work horses undertake has been shown to influence both personality and welfare state. An experimental study performed on horses living in the same conditions, belonging to one breed and of the same sex, but differing only by the type of work undertaken, revealed that the reactions to emotionality tests strongly depended upon the type of work horses were used for and not on the type of work they had been initially selected for [25]. The prevalence and types of stereotypies observed differed according to the type of work they had been used for at least one year [26]. Although it is well known that stereotypic behaviours may emerge in relation to a variety of factors (*e*.*g*., weaning, feeding, housing conditions; reviewed in [27]), in the present case only the type of work emerged as a determinant of these differences as all environmental factors were controlled and some horses came from the same breeding farms. These findings converge with those of several questionnaire-based or observational epidemiological studies suggesting an impact of the type of work on the emergence of stereotypic behaviours [28–30] or back disorders [31, 32]. Since horses with back disorders show a lower/different attentional engagement [33, 34] and stereotypic horses different cognitive functioning from non-stereotypic horses (review in [35]), these problems may reflect a secondary influence of the type of work on cognitive performance such as attention. It has also been argued that repeated stress at work may be associated with learned helplessness [36], and/or lead to apathetic/depressed states (*i*.*e*., unreactive/inattentive to sensory stimulations) in working horses at their place of work [37] or in their home stall [38–40].

As in humans or dogs, there may be a more direct impact of working conditions on attentional skills. For example, Hausberger et al. [41] suggested that the lower performances of high school horses in an instrumental learning task could be explained by the fact that these horses have to perform highly sophisticated exercises leading to little freedom on how to behave at work, and less opportunity to learn to learn. In this case, horses have to concentrate solely on the rider’s orders without being influenced by environmental factors. One could expect that outdoor activities such as eventing, that requires adapting the jumping style to each natural obstacle, or recreational outdoor riding, that requires paying attention to both riders’ demands and environmental characteristics, may require a more divided type of attention.

In the present study, we hypothesized that horses used for different types of work would demonstrate different attentional characteristics. In order to test this hypothesis, we exposed sixty-two horses to the visual attention test (VAT), that had proved useful for characterizing horses’ individual attention characteristics and for predicting horses’ attention and learning performance in an operant task as well as in a working task [20]. It is generally difficult to disentangle the effect of life conditions or intrinsic characteristics from those of the type of work *per se* [30]. Since it is very rare to have a large sample of horses working in different disciplines on the same site [26], we studied four different working populations, living in two different types of conditions: *i)* two populations living under restricted conditions (*i*.*e*., individual stalls) but used either for instruction (riding school) or competition (show jumping or eventing); *ii)* two populations living under ‘naturalistic’ conditions (*i*.*e*., group in pasture) but either used for recreational riding (outdoor leisure riding) or not ridden. In view of the literature, we predicted that individual attentional characteristics would be influenced by the type of work they are trained for and potentially less by intrinsic characteristics such as sex, breed or age. Notably, it is commonly said that stallions are less attentive than mares but previous studies revealed no significant difference [22, 42]; horses’ breeds influence cognitive performances which could be related to attention characteristics [41, 43]: for instance, non-warmblood horses seemed to have better learning performance [44]; attentional decrease could be associated with ageing [45] but previous studies revealed no significant difference [20] or no linear decrease [42] with age.

## Materials & methods

### Ethical note

This study was approved by the French national ethics committee (Animal utilization protocol number: 33, 12-2013-12). All Experiments complied with current French laws related to animal experimentation and were in accordance with the European directive 2013/118/CEE (decree n°2013–118 of 1 February 2013 and its five implementation orders (JO of 7 February 2013), integrated in the Code rural and the Code of the maritime fishing under n° R.214–87 a R.214-137). This experiment involved only behavioural observations and non-invasive contacts with horses. Animal husbandry and care were under management of the riding school staff, the ‘Ecole Nationale d’Equitation’ staff, the staff of the research station in Chamberet, the staff of the research station in Paimpont and a private owner.

### Subjects

#### Overall conditions

Sixty-two horses, aged 5 to 22 years ($\bar{X}$± SEM = 11.0 ± 0.6 years old) were tested (between 2012 and 2014) in 4 different sites where two modalities of management prevailed:

- Restricted conditions of life for the sites 1 and 2: all horses were kept singly in 3 m * 3 m individual stalls that were mostly straw-bedded (only 5 horses from site 2 had wood shavings). Some contact with the closest neighbour was possible through bars between stalls in site 1 but not site 2. Water was provided *ad libitum* and hay was provided once (site 1) or twice (site 2) a day. Horses were fed commercial pellets three times a day.
- Naturalistic conditions of life for the sites 3 and 4: all horses spent most of their time in groups in pastures and were fed grass outside winter where they received hay and pellets (site 4). They were occasionally kept in stalls for some hours or days for work, care (site 3) or parturition (site 4), which allowed testing them easily in a stall.

#### Populations and working conditions

Site 1: riding school: 27 horses (6 mares and 21 geldings) from the same French riding school were tested. They were between 5 and 20 years old ($\bar{X}$± SEM = 12.7 ± 0.9) and 74% of the horses were French Saddlebreds. One caretaker was responsible for all horses. Horses worked in riding lessons for 6–10 hours a week, with at least 1 free day each week when they were in outdoor paddocks. Riding lessons involved experienced and novice children and teenagers and were mainly related to indoors activities with mostly tight reins and tensed type of riding [32].
Site 2: sport horses: 17 horses (7 mares, 10 geldings), aged 7 to 12 years ($\bar{X}$± SEM = 8.1 ± 1.6) were tested at the ‘Ecole Nationale d’Equitation’ of Saumur (France). They were from two different breeds: French Saddlebreds (N = 13, 76%) and Anglo Arabian (N = 4, 24%). The horses were used for either jumping (Jump, N = 10) or eventing (Event, N = 7) competitions. They had arrived at the age of 4–5 years old, and had not been selected for any other work than jumping initially. Some of the horses in the two discipline groups were half-siblings (same sire). One caretaker was responsible for a group of 8 to 10 horses and one experienced rider for a group of 3 to 4 horses. Horses were ridden for an hour daily (leading to 5 to 7 hours a week). In order to test further the relationship between attentional characteristics and type of work, we obtained the competition performance index for the eventing horses (*i*.*e*., individual score of performance in competition since the beginning of each horse career, taking into account the competition’s discipline, level and the rider, made available on the website of the French National Studs; [46]).
Site 3: recreational horses: 6 horses (2 mares, 2 geldings and 2 stallions) were tested. They were between 9 and 22 years old ($\bar{X}$± SEM = 15.1 ± 1.8) and belonged to a variety of breeds (50% unregistered horses, 50% crossbreds: French Saddlebred, Quarter horse, Spanish). They were occasionally ridden outdoors with long reins and low hands in varied areas.
Site 4: breeding horses: 12 broodmares, aged 5 to 17 years old ($\bar{X}$± SEM = 8.9 ± 1.1), non-pregnant and from Anglo-Arabian (83%) and French Saddlebred (17%), breeds were tested. All horses were born and had been raised in the same breeding farm under similar management (*i*.*e*. the ‘Station Expérimentale des Haras Nationaux’ (SEHN), ‘Institut Français du Cheval et de l’Equitation (IFCE)’, Chamberet, in France). They were kept in large social groups of females (30 mares) in large pastures from June to October and indoors in large stalls from November to April. These mares had never been ridden.

### Visual attention test (VAT)

#### Test procedure (see also [20])

All tests were performed by the same experimenter (CR). Horses were brought in a familiar stall. Their spontaneous visual attention was tested by using a standardized circular moving visual stimulus (Ø1cm). A green light issued from a laser pointer (Laser point, PEARL^®^) was projected on each horse’s stall doors during 5 minutes.

The experimenter entered the stall, approached the horse, equipped it with a halter and a rope and then placed it gently in front of the closed stall door. The horse was then left unrestrained (the rope being placed over its neck) and hence was able to move freely. During a ‘human presence habituation phase’ of 2 minutes, the experimenter stood motionless in the middle of the stall at 1m distance from the stall door (facing it), on the left side and at the shoulder level of the horse. Then, during the test phase, the experimenter stood at the same location, arms along the body, limiting movements to the small wrist movement when displaying the stimulus. As soon as the horse directed its head towards the lower part of the stall door, the experimenter projected the visual stimulus for 5 minutes (*i*.*e*. 300 seconds), straight ahead from the horse, on the stall door (1 meter from the horse) with repeated circular clockwise and 50-cm long vertical and horizontal movements, following a predetermined order [20]. If the horse wanted to move, the experimenter did not restrain it. Each horse was tested once and individually.

#### Data recording

**Fig 1:**
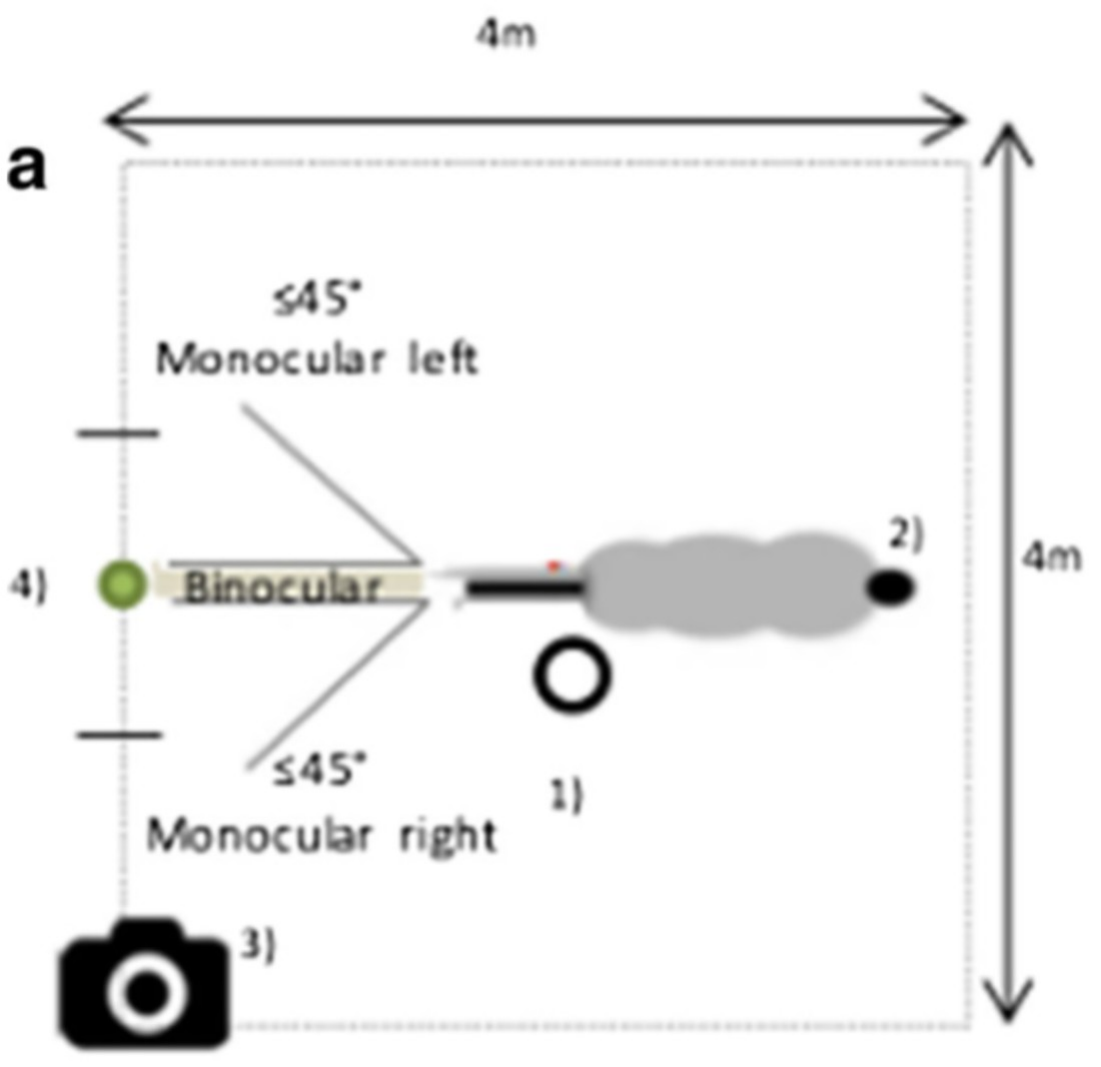
Diagram of the experimental setup of the visual attention test (VAT): Horses’ position in the stall during the VAT: 1 the experimenter, 2 the horse, 3 the camera; and 4 the stall door where the visual stimulus was projected. Binocular gaze: the horse faced the visual stimulus with head (*i*.*e*. mid line of the head) and both eyes oriented towards it. Monocular gaze: the horse faced the head at less than 45° to the right or to the left towards the visual stimulus. Angles were evaluated with respect to the stall horizontal plan and to the mid stall door wall where the stimulus was projected.

All tests were videotaped using an axis M1054-W camera^®^ (lens 2.8 mm; horizontal field view: 84°) mounted on the top of the left stall corner above the place in which the stimulus appeared (*i*.*e*. 3 meters high; 3 meters from the stall door). Since it was set sideward, the stimulus and horses’ head and neck angle as well as gazes could be recorded (Fig 1) [20]. The data were transcribed later by 3 observers (CR, MS, MHO), each analysing one site separately and hence, being without any expectation according to work influence; inter-observer reliability: Spearman rank order correlation *r* = 0.86). Horses’ expressions of forward attention are characterized by anteriorly directed orientation for reception of visual cues: their ears are upright and oriented forwards, their eyes look forwards and they appear to enhance their binocular visual field, and their neck and head angle is adjusted to facilitate reception by their sensory organs [47, 48].

Using continuous focal sampling [49], the total amount of time spent gazing at the stimulus was recorded frame by frame (frame, 0.02s). Horses have laterally placed eyes with a small (60–80°) binocular field of vision and almost complete (80–90%) decussation of the optic nerves, suggesting that behavioural asymmetries reflect asymmetries in hemispheric activation [50]. Binocular gaze towards the stimulus was defined when the horse faced the head (mid line of the head, between both eyes) and both eyes were oriented forwards towards the visual stimulus. Monocular gaze towards the stimulus was defined when a horse faced the head (mid line of the head, between both eyes) ≤ 45° to the right or to the left towards the visual stimulus. Angles were evaluated with respect to the stall horizontal plan and to the mid stall door wall where the stimulus was projected. A gaze was defined if the horse had at least one moment of ≥ 1 sec of gazing fixedly [51]. The ‘beginning’ or ‘end’ of a gaze was defined when the horse’s head started to move into or out of the ≤ 45° zone, respectively [52].

#### Data analysis

The following temporal and structural data, based on [20], were used to characterize horses’ attention:

1. the *reaction time* (in seconds), defined as the time between the first occurrence of the stimulus and the horse’s first gaze at the stimulus. Reaction time was considered to estimate attentional capture. Subjects that did not gaze at the stimulus during a test were given a maximum reaction time score of 300 seconds.
2. the *type of attention*: a) the *overall attention* characterized by all gazes and associated with horse’s body movements (neck, head, ears and eyes). Both monocular (the use of one eye visual field with horse’s head oriented less than 45° to the right or to the left (see above) of the stimulus [53] and binocular gazes (horse’s head and both eyes oriented forwards towards the visual stimulus, were recorded; b) the *fixed attention* characterized by the exclusive use of the binocular visual field associated with a total fixity of the horse’s body (including neck, head, ears and eyes) and both ears pointed towards the stimulus.
3. The *temporal parameters* of the respectively overall and fixed visual attention were estimated by the following scores:
  - *duration (in seconds) of each gaze sequence* leading to an overall sequence mean duration score.
  - *total duration (in seconds) of all gazes* at the stimulus (*i*.*e*. sum of durations of all gazes during the 5-minute test)
4. The *structure* of the respectively overall and fixed visual attention was estimated by the following scores:
  - *number of gaze sequences* (*i*.*e*. number of times when horses gazed at the stimulus during the 5-minute test),
  - an *index of attention fragmentation* calculated as the ratio between the number of gaze sequences and total duration of gazes during the 5 minutes’ test *(number/seconds)*. The index gives an idea of alternating attention abilities (*i*.*e*. the ability to shift the focus of attention). The higher the index, the more the attention was fragmented.

### Statistical analyses

In order to analyse how attention variables correlate with one another and how it is related to individual attention characteristics, we used a Principal Component Analysis (PCA) followed by a Ward hierarchical classification analysis (HCA) as a descriptive but informative multifactorial analysis. PCA and HCA were performed on reaction times, total duration of attention, number of attention sequences, duration and fragmentation of these sequences. We performed PCAs with scaled-centered variables. We retained principal components (*i*.*e*., variables that are constructed as linear combinations of the initial variables, with the first principal component explaining the largest possible variance of the dataset) with eigenvalues greater than 1 (Kaiser–Guttman criterion, Table 1 in S1 Appendix). PCA and HCA describe the relative position of each individual according to attention characteristics.

We assessed the influence of intrinsic (*i*.*e*. age, sex, breed) and extrinsic (*i*.*e*. living and working conditions) factors on attention characteristics using linear models (LM). We ran separate LM for each attention characteristic as a dependent variable, age was included as a covariate, sex and breed were included as fixed effect and the type of work nested in living condition (categorical explanatory variables) were also included as fixed effect. We assessed all possible two-way interactions between the covariates and explanatory variables and excluded them stepwise if they proved non-significant based on log-likelihood ratio tests. Thus, we stepwised models to get to a simpler model proved to have a better fit and based on AIC (*i*.*e*., the Akaike information criterion; lrtest’, ‘lmtest’ package; [54]).

We tested the relationship between attention characteristics and score in competition in sports horses using Spearman rank correlation. We tested the proportion of horses showing short or long attention duration sequences from different study sites using Fisher exact test.

All statistics were performed with R v. 3.6.1 [55] (The R foundation for statistical computing, http://www.r-project.org/, Team 2019). The significance level was set at 0.05. Descriptive statistics are reported as means and standard error of mean (SEM). PCA was performed using *FactomineR* package [56]. Independence and homogeneity of variances of the models were assessed by inspection of fitted values residuals using the *plotresid* function in *RVAideMemoire* package [57]. Linear models were constructed using the lm function in *lme4* package [58] and statistical tests were performed using the Anova function in *car* package [59]. *Post hoc* tests were performed using the *emmeans* function in the *emmeans* package [60], along with the contrast pairwise comparison function (*emmeans*, “pairwise”) using t-test with a Tukey correction.

## Results

### Horse’s attention during the VAT

All horses were attentive at least once towards the visual stimulus. The reaction time ranged from 0.1 to 168.0 seconds (15.1 ± 3.8s) and total duration of attention varied between 3.0 and 273.0 seconds (72.9 ± 8.9s) with the number of attention sequences varying between 1.0 and 28.0 (8.1 ± 0.7) and a mean duration of sequences between 0.9 and 43.0 seconds (9.4 ± 1.2s). The fragmentation index (ratio of the number of attention sequences/total duration of attention) varied between 0.02 and 1.1 (0.2 ± 0.02 nb/s). Fixed attention duration within the 5 minutes of test varied from 0.0 to 94.5 seconds (19.4 ± 3.2s) including a number of fixed attention sequences from 0.0 to 22.0 (4.2 ± 0.6) which duration ranged from 0.0 to 13.2 seconds (3.2 ± 0.4s). The fragmentation index of fixed attention varied between 0.0 and 1.0 (0.3 ± 0.1 nb/s).

### Factors of variation of attention characteristics

**Fig 2:**
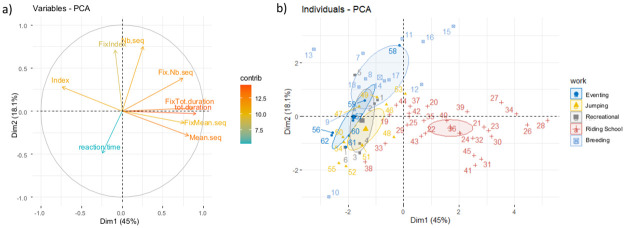
Graphical results from the Principal Component Analysis (PCA). a) Variables factor map obtained from the PCA on attention characteristics. A small angle between variables implies positive correlation, a large one suggests negative correlation, and a 90° angle indicates no correlation between two characteristics. Dimension 1 corresponds to the first component and opposed the total durations of both overall and fixed attention to the index of fragmentation. Dimension 2 corresponds to the second component and opposed the number of overall attention sequences and the index of fragmentation to the reaction time and mean duration of sequences of overall attention; Variables with high contribution to the dataset variance are in red/orange whereas variable with low contribution are in blue; b) Factor map obtained from the PCA illustrating the distribution of horses according to their attention characteristics (each number correspond to a horse). Colors and ellipses illustrated the type of work (Eventing: N = 7; Jumping: N = 10; Recreational: N = 6; Riding school: N = 27; Breeding: N = 12). Barycenter for each work type are shown.

The first two components of the PCA performed on all subjects’ attentional characteristics accounted for 63% of the variance and the first three ones to 74% (dimensions 1: 45%; dimension 2: 18%; dimension 3: 11%; Fig 2a; Table 1 in S1 Appendix). Dimension 1 was explained by the total durations of both overall and fixed attention opposed to the index of fragmentation. Individuals characterised by long duration of overall and fixed attention and low attention fragmentation are on the right (Fig 2a and 2b). Dimension 2 was explained by the number of overall attention sequences and the index of fragmentation opposed to the reaction time and mean duration of sequences of overall attention. Individuals characterised by higher number of attention sequence and fragmentation are near to the top (Fig 2a and 2b; Table 2 in S1 Appendix). Finally, dimension 3 was explained by reaction time opposed to the index of fragmentation (Table 2 in S1 Appendix). The distribution of horses in the PCA, although based solely on individual attentional characteristics, appeared to be highly related to the type of work whereas life conditions, sex, breed or age were rather evenly distributed (Fig 2b). Thus, the riding school horses appeared, along dimension 1, to have opposite characteristics to those of all other populations, including those living in similar conditions (single stall housing: sport horses: Eventing and Jumping). Axis 2 opposed the breeding horses to both sport and recreational horses, despite the latter living in similar conditions. The difference was more important with the sport horses practicing jumping. The recreational horses appeared intermediate between the jumping and eventing sport horses on axis 2 but separated from all other populations on Axis 3 (reaction time).

**Fig 3:**
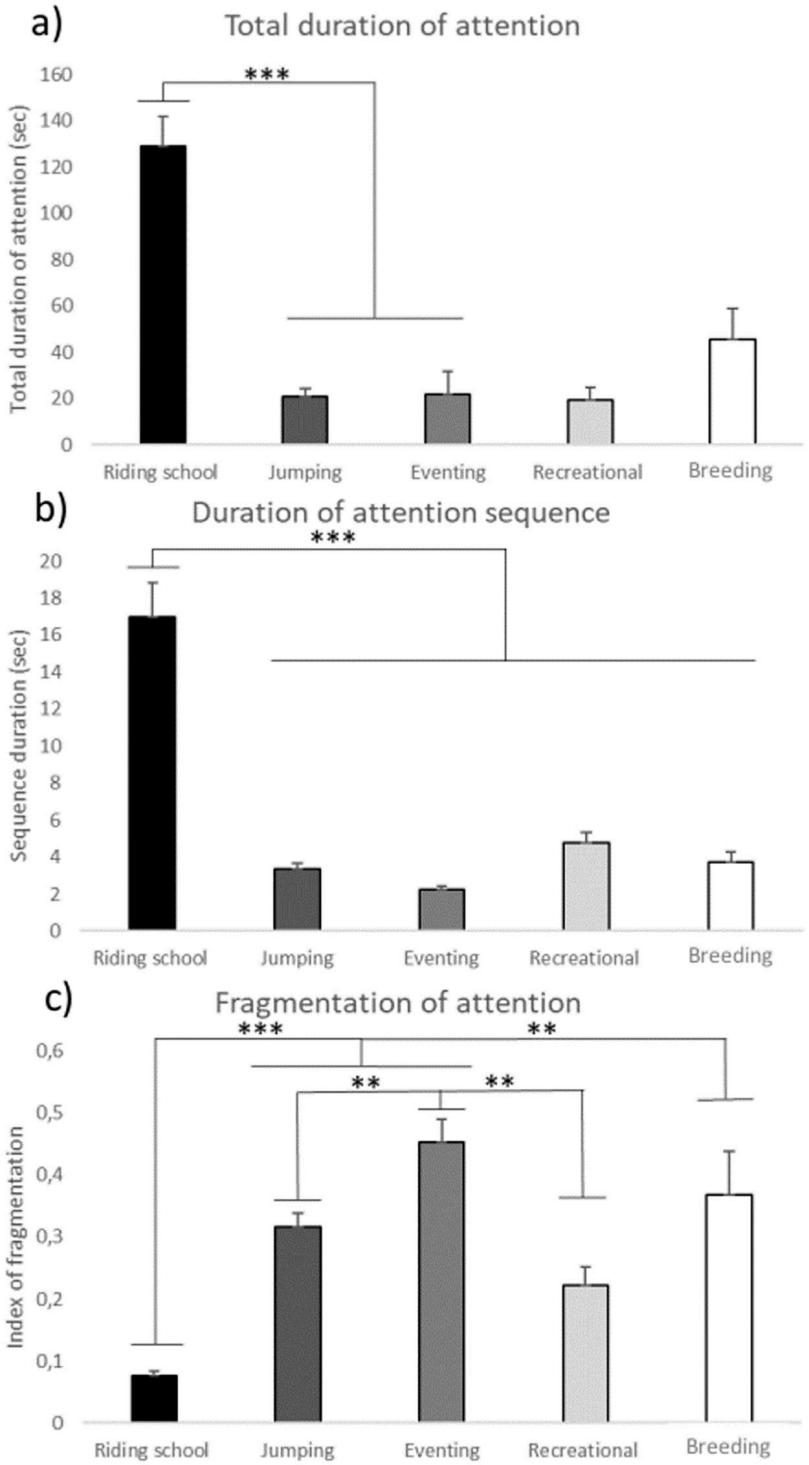
Mean ± SEM of attention characteristics according to the type of work. a) total duration of overall attention (in seconds); b) average duration of overall attention sequences (in seconds); c) fragmentation of overall attention (ratio number of attention sequences / total duration of attention); Differences according to the type of work (site 1, riding school horses N = 27; site 2, sport horses N = 17 with jumping horses N = 10 and eventing horses N = 7; site 3, recreational horses N = 6; site 4, breeding horses, N = 12). LM with *post-hoc* t-test with Tukey correction, ** P < 0.01; *** P < 0.001.

Linear models confirmed these differences: all attention characteristics with the exception of 2 (reaction time, number of sequences) were significantly influenced by the type of work (Table 1). The attention characteristics were not significantly influenced by horses’ sex, age and breed (Table 1). Riding school (RS) horses clearly differed from most other populations with longer total durations of overall attention (LM, N = 62; F = 12.69, P < 0.0001; *Post Hoc* t-test: N_riding school_ = 27, N_Event_ = 7, N_Jump_ = 10, N_Recreational_ = 6, N_breeding_ = 12; RS/Event: t_49_ = -4.26, P = 0.0008; RS/Jump: t_49_ = -5.05, P = 0.0001; but RS/Recreational: t_49_ = -2.58, P = 0.089, RS/Breeding: t_49_ = -2.76, P = 0.059) (Fig 3a) and longer durations of overall (LM, N = 62; F = 12.47, P < 0.0001; *Post Hoc* t-test: RS/Event: t_49_ = -4.10, P = 0.001; RS/Jump: t_49_ = -4.91, P = 0.0001; RS/Recreational: t_49_ = -3.04, P = 0.029, RS/Breeding: t_49_ = -2.90, P = 0.042) (Fig 3b) and fixed (LM, N = 62; F = 3.36, P = 0.026; *Post Hoc* t-test: RS/Event: t_49_ = -2.86, P = 0.047; RS/Jump: t_49_ = -1.89, P = 0.334; RS/Recreational: t_49_ = -1.82, P = 0.371, RS/breeding: t_49_ = -2.72, P = 0.064) attention sequences. Thus, they also showed less fragmentation of overall attention (LM, N = 62; F = 13.48, P < 0.0001; *Post Hoc* t-test: RS/Event: t_49_ = 5.74, P < 0.0001; RS/Jump: t_49_ = 4.47, P = 0.0004; RS/Recreational: t_49_ = 1.21, P = 0.746, RS/Breeding: t_49_ = 4.06, P = 0.002) (Fig 3c). Interestingly, eventing horses presented also more fragmentation of overall attention than the jumping and recreational horses (*Post Hoc* t-test: Event /Jump: T_10_ = 3.21, P = 0.009; Event/Recreational: T_10_ = 4.99, P = 0.0004). Recreational horses, breeding horses and sport horses showed durations of fixed attention sequences between 1 and 2 seconds, whereas more than 50% of riding school horses showed fixed attention sequences lasting more than 4 seconds (Fisher exact test, P < 0.05).

**Table 1:** Attention characteristics and influence of intrinsic (sex, age, breed) and of living condition and type of work.

Variables	*F*	*Df*	*P*
*Reaction time*			
Sex	3.74	1	0.058
Age	0.20	1	0.652
Breed	1.37	6	0.244
Living condition	0.96	1	0.421
Type of work	0.80	3	0.498
*Total duration*			
Sex	1.13	1	0.294
Age	0.57	1	0.454
Breed	1.50	6	0.198
Living condition	0.14	1	0.706
Type of work	9.74	3	**<0.0001**
*Number of sequences*			
Sex	0.29	1	0.594
Age	0.36	1	0.550
Breed	0.50	6	0.804
Living condition	0.68	1	0.410
Type of work	0.72	3	0.541
*Fragmentation index*			
Sex	0.02	1	0.880
Age	0.01	1	0.992
Breed	0.19	6	0.979
Living condition	0.11	1	0.740
Type of work	13.48	3	**<0.0001**
*Sequence duration*			
Sex	0.97	1	0.329
Age	0.61	1	0.439
Breed	2.21	6	0.058
Living condition	0.02	1	0.876
Type of work	12.47	3	**<0.0001**
**Fixed attention**			
*Total duration*			
Sex	0.09	1	0.758
Age	4.50	1	0.053
Breed	1.18	6	0.332
Living condition	0.01	1	0.929
Type of work	2.74	3	0.053
*Number of sequence*			
Sex	0.08	1	0.781
Age	0.83	1	0.365
Breed	0.50	6	0.805
Living condition	0.39	1	0.528
Type of work	3.25	3	**0.029**
*Fragmentation index*			
Sex	0.04	1	0.841
Age	0.67	1	0.417
Breed	1.29	6	0.281
Living condition	0.57	1	0.449
Type of work	1.98	3	0.129
*Sequence duration*			
Sex	0.53	1	0.471
Age	1.34	1	0.252
Breed	1.59	6	0.169
Living condition	0.02	1	0.886
Type of work	3.36	3	**0.026**

Type of work influence was nested within the living condition (**bold value:** P < 0.05).

**Fig 4:**
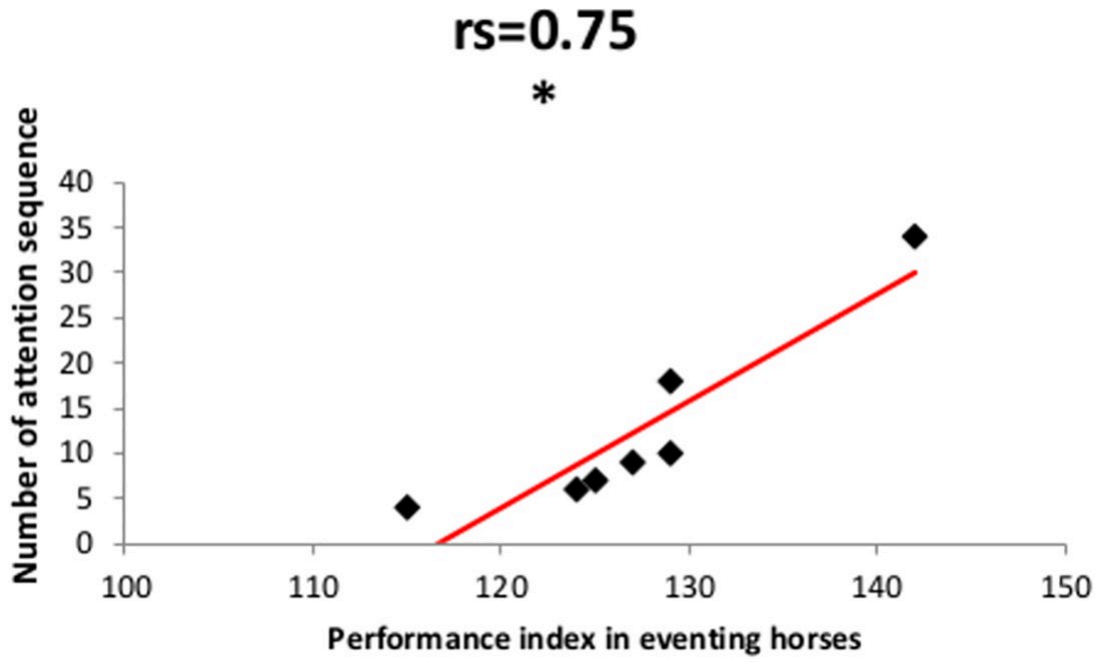
Correlation between the performance index *i*.*e*., individual score of performance in competition since the beginning of each horse’s career, for eventing horses and the number of overall attention sequences towards the visual stimulus during the 5 min of the VAT test, Spearman’s rank correlation test, N = 7, * P <0.05.

We looked further at the relationship between attentional characteristics and type of work by examining the potential link with performance in eventing sport horses. Interestingly, despite the low sample, we found a positive correlation between the number of overall attention sequences expressed by eventing horses during the test and their index of performance (Spearman correlation test, N = 7, *rs* = 0.75, P = 0.048) (Fig 4). Thus, the number of attention sequences during the 300 sec. of test, and hence the fragmentation, appeared to be a potential indicator of horses’ competition performance.

## Discussion

Our study, based on several populations of domestic horses, showed that intrinsic factors such as age, sex, and breed did not influence individual attentional characteristics in a visual attention test whereas the type of work appeared as a major factor of influence. Riding school horses showed longer sequences of overall attention and a longer total duration of attention during the test and hence, less fragmentation of attention than the other horses working in different disciplines, including sport horses living in the same housing conditions. Moreover, horses living in the same conditions but trained either for show jumping or eventing showed differences in attention characteristics: eventing horses were characterized by more fragmentation of attention compared to jumping horses. This attention characteristic was positively correlated to eventing horses’ performance in competition. Recreational and breeding horses showed an intermediate attentional profile. The observed individual differences in attention according to the type of work revealed in the present study were remarkable as they were tested in a non-work-related context. In view of the results, we propose three hypotheses: 1) a possible selection of animals presenting suitable behaviour (involving attention characteristics) for the type of work they are used for, 2) a direct impact of training and working on the development of attentional characteristics, 3) an indirect impact of work on attentional characteristics through its influence on horses’ welfare state.

Individual differences in attentional characteristics have been described in different species. In working dogs, studies have attempted to characterize the attentional skills required to choose the most appropriate dogs for the type of work undertaken [15]. In horses, the practice of a particular discipline requires not only that the physical capacities but also the personality and the cognitive abilities be adapted to the type of discipline carried out [61]. In a previous study, we described individual differences in attention that were stable over 6 months at least, but these were unridden horses kept in the same living conditions all this time [20]. Further research is needed here to more thoroughly examine potential intrinsic individual attentional characteristics. It is interesting however, that we did not find any influence of intrinsic factors such as breed, age or sex. For example, attention is supposed to decline in normal ageing in humans and non-human species [62]. However, it has been shown that the relationship between attention and age is not linear: selective attention performance is peaking at middle age in horses [42], dogs and humans [63]. Attention can also be modulated by different factors, such as chronic pain, which are not related with age [33, 64]. The absence of influence of intrinsic factors also suggests that environmental factors such as work may have more impact on attentional characteristics. Moreover, even in horses of similar breeds living in the same conditions, differences occurred between horses working in closely related types of work like jumping or eventing. Among eventing horses, those with more fragmented attention during the visual attention test had higher performance index score in competition, which aligns to the positive correlation between attention shifting and updating and performance in elite soccer players [13]. One explanation could be that horses initially more prone to attention shift and attention fragmentation would have been selected for eventing discipline. Indeed, horses’ selection is based on competitive performance directly at the individual level or by including ‘bloodlines’ with the performance of related individuals [46]. But previous studies exploring horses’ eventing performance reported that the most important predictors of eventing performance in competition were ‘environmental’: non-genetic repeatable contributors to phenotypic variance, such as training and nutrition, followed by the rider experience and horse genetics [65, 66]. Moreover, in the present study, all sport horses (and thus their bloodlines) had initially been selected for jumping and several half siblings (same sire) were working in both types of work (eventing and jumping). In any case, French saddlebreds in riding schools differed greatly from their sport counterparts, despite all living in similar conditions. Although, it is impossible at that stage to eliminate the hypothesis of an active choice by riders of animals presenting attention characteristics adjusted for the type of work they were used for, it seems more likely that the individual differences observed may result from external factors.

One other complementary explanation therefore could be that the work demands on cognitive processes such as attention differs between disciplines, as observed in humans [7, 10] and other animals [16, 17]. Our results converge with those on human sports athletes. elite basketball and soccer players show better performances in attentional shifting tasks [11, 13]. In other animals such as dogs, untrained pets individuals and dogs trained either for agility, search or rescue showed differences in attention characteristics towards humans during a problem-solving task: agility dogs showed more gaze alternation between the person and the apparatus than rescue and pet dogs suggesting an influence of individual training experiences [17]. In the present study, eventing horses for example have to face, at high speed, many environmental stimuli during the cross-country event (*e*.*g*., different jumps, approaches, distances) and would be more required to switch their attention and thus to quickly change focus of attention. Moreover, eventing includes also show jumping and dressage events [66], which both require specific training and additional (and maybe complementary) attentional abilities. The latter point could partly explain the difference between eventing and jumping horse’s attention characteristics, observed here despite the low sample and exactly similar living conditions. Jumping horses have to face less environmental stimuli, training and competition occur mostly in dedicated arenas. Unridden horses were also characterized by high attention fragmentation, which may also be related to the fact that grazing horses have to divide their attention between selecting food and watching for potential dangers/stimulations in the environment [47]. Interestingly, recreational horses, despite living in very different conditions from sport horses, showed a close pattern of attention. This similarity could be due to their riding in varied environments that require similar attention shifts, but their lower reaction time compared to sport horses may be related to the much quieter type of riding that may not require rapid decision taking as at the fast paces required by jumping or eventing. Other factors of influence on attention, to which we did not have access, are for example the conditions of earlier training of the horses: the predominant use of positive *versus* negative reinforcement during training may also strongly influence attention patterns and work memories [22, 35, 67].

Finally, the type of work may also exert influences on chronic welfare and thus on cognitive processing (*e*.*g*., [35]). In humans, cognitive alteration is consistently reported in depressed patients, and hypothesized to be involved in the onset and/or maintenance of this disease [68]. In animals, apathetic states are associated by a lack of interest towards the environment and immobility associated with fixed staring [69]. For example, dogs involved in animal-assisted intervention have to calmly tolerate sometimes invasive interactions and may experience repeated stress that transforms into apathetic states, associated with a decrease of overall attention towards social and non-social environment [70]. Several studies have described apathetic states in working horses [38, 40] and in close relationship with working conditions [37, 71]. In Fureix et al.’s [38] study, moments of apathetic state in horses were characterized by immobility of body, head and neck, as well as long gazing behaviours lasting up to 97 seconds (no visible eye movements) which differed from moments of calm immobility in non-apathetic horses with eye and head movement and lasting at most 20 seconds [38]. Also, the prevalence and type of stereotypic behaviours horses perform in their home environment is influenced or associated with the type of work the horses are used for [26]. These abnormal behaviours under their different forms, are associated with atypical cognitive processes [72], that could be related to perseveration [73] or lower learning abilities in an instrumental task ([74], review in [35]).

There are different ways the type of work may exert chronic influences on the welfare state of horses. For example, work may be a source of stress through the inhibition of behavioural expressions associated with emotional states, or the equipment or riders’ actions that can induce discomfort or even pain in horses [26, 28, 75–77]. For example, stereotypic behaviours observed may emerge from pain at work that becomes chronic with repetitions [26, 78]. Finally, work may induce back disorders, and hence chronic pain (review in [31]), as different studies have shown that the prevalence, location and type of back problems differed between disciplines of western riding [79], between race- and sport- horses [80] and overall were most prevalent in riding school horses [81, 82]. Attentional engagement (*i*.*e*., alertness that allow individuals to process information from the environment *e*.*g*., [83]) is known to be affected by chronic pain in rats [84], humans [85], and by chronic back problems in horses [33]. In horses, Lesimple et al. [28, 31, 32] found that, in beginners’ lessons, riding technique (*i*.*e*., high hands, tight reins), influenced by the teacher’s pedagogy, was one of the most influential factors in the prevalence of back problems and stereotypies in riding school horses. This problem remains mostly undetected, and hence, a source of repeated stress at work [80, 86]. A recent study using a portable electroencephalography system has shown an association between back health state and EEG power spectra [34]. Horses with a healthy back showed more slow waves and especially theta waves, known to be associated with calm attention [87] than horses with tension along the spine, characterized by more fast waves [87]. This is in accordance with the finding that horses with a healthy back spent more time observing the environment (*i*.*e*., in calm attention) than horses with back problems [33]. We did not have measures of the welfare state nor back state here but the fact that riding school horses clearly differed from all the other tested populations is interesting, in view of the existing literature showing that such horses are more at risk of developing back problems. They showed longer sequences of attention, longer total duration of attention and hence less fragmentation. This could reflect a higher prevalence of depressive-like horses in this population, as such horses show longer gazing bouts, although the durations of gazes during the VAT were clearly shorter than during the apathetic bouts described in [38]. Paying little attention or paying attention for too long could correspond to an attention deficit or a difficulty in filtering out irrelevant information from the environment (*e*.*g*., in dogs: [63]). One alternative and not exclusive explanation could be the absence of environmental stimulations in riding school horses’ daily lives with most time spent in indoor stalls and work in an indoor arena. The visual stimulus could then be a source of prolonged interest. However, jumping horses, also housed indoors and ridden in dedicated arenas showed a very different pattern of attention. Finally, riding school horses were also those that worked the most (up to 10h a week) in a ‘routine’ way, which could have led to a ‘mental fatigue’ that is strongly related to attention alteration in humans [7]. Repeated actions (such as the tasks repeated 10 hours per week) may however require less cognitive resources *e*.*g*., [88, 89] and a lower risk of mental fatigue. However, it is not sure the tasks performed by riding school horses are more repetitive (various riders’ levels, basic instruction to somewhat advanced jumping or dressage) than those performed by jumping horses which only task at each training is often to jump artificial obstacles. Only further studies could provide the answer. The possibility that riding school horses have a higher lack of control over all aspects of their lives, leading to a higher prevalence of depressive states (25% in [38]) may also explain their lack of attention fragmentation. Interestingly, here, the unridden horses appeared intermediate between these riding school horses and sport horses, with less fragmentation of attention than the latter but more than the riding school horses.

The results of this study therefore open new lines of thought on the determinants of animal cognition and its plasticity. Despite limitations, such as the sample size for some populations and the possible bias due to the presence of the experimenter during the tests, the data clearly indicate differences in individual attention characteristics between populations of horses used for different type of work. Moreover, the presence of a human during the test may, in the present case, be favourable to predict the working situation which is strongly associated with human presence [90, 91]. Further studies (with or without the presence of an experimenter) on larger samples and integrating other aspects such as back health, welfare indicators and modalities of training should help understanding further this interrelationship between working conditions and cognition.

## Supporting information

S1 Appendix (DOCX)

S1 Data (XLSX)
