# Supplementary material for: Horses’ attentional characteristics differ according to the type of work
Source: PLoS One. 2022 Jul 25;17(7):e0269974. doi: 10.1371/journal.pone.0269974 (PMC9312386; doi:10.1371/journal.pone.0269974)
Supplement: S1 Appendix — (DOCX) [file pone.0269974.s001.docx]

**Appendix 1**

Table 1: Eigen value from PCA

| Dimension | Eigen value | Variance % | Cumulative variance % |
| --- | --- | --- | --- |
| Dim.1 | 4.05109397 | 45.0121552 | 45.01216 |
| Dim.2 | 1.62531348 | 18.0590387 | 63.07119 |
| Dim.3 | 1.03622392 | 11.5135991 | 74.58479 |
| Dim.4 | 0.72551062 | 8.0612291 | 82.64602 |
| Dim.5 | 0.60263074 | 6.6958971 | 89.34192 |
| Dim.6 | 0.47770036 | 5.3077817 | 94.6497 |
| Dim.7 | 0.39480716 | 4.3867463 | 99.03645 |
| Dim.8 | 0.0647868 | 0.7198534 | 99.7563 |
| Dim.9 | 0.02193295 | 0.2436994 | 100 |

Table 2: Variable contribution (**bold** value showed variable with high contribution).

|  | Dim.1 | Dim.2 | Dim.3 | Dim.4 | Dim.5 |
| --- | --- | --- | --- | --- | --- |
| Reaction time | 1.52 | 14.68 | **31.47** | 49.38 | 1.42 |
| Total duration | **20.29** | 0.04 | 4.40 | 2.43 | 9.99 |
| Number of sequence | 1.65 | **34.47** | 7.26 | 0.53 | 41.21 |
| Fragmentation index | 13.47 | 4.80 | 7.02 | 2.32 | 0.77 |
| Sequence duration | 16.28 | 4.91 | 12.91 | 4.01 | 2.01 |
| Fixed: Nb of sequence | 13.72 | 8.76 | 10.38 | 0.66 | 3.06 |
| Fixed: Total duration | **18.70** | 0.08 | 12.59 | 3.60 | 7.33 |
| Fixed: Fragmentation index | 0.19 | **31.07** | 8.81 | 37.03 | 20.92 |
| Fixed: Sequence duration | 14.19 | 1.20 | 5.15 | 0.05 | 13.30 |
